# Lessons from the past to understand the future

**DOI:** 10.1111/ene.16270

**Published:** 2024-03-05

**Authors:** Didier Leys, Claudia Sommer, Barbara Borroni, Andrew Chan, Mark Edwards, Alessandro Tessitore, Kristl Vonck

**Affiliations:** ^1^ European Academy of Neurology Vienna Austria

For the 30th anniversary of the *European Journal of Neurology*, we publish a special issue entitled “Lessons From the Past to Understand the Future.”

This special issue includes articles concerning the organization of neurology in Europe and its evolution over time, in all its components: patient care, teaching, and research. These articles show how two societies created at the end of the 1980s merged into a single entity in 2014, the *European Academy of Neurology*, retaining the principles of the two parent societies: representing national neurological societies (principle of the former *European Federation of Neurological Societies*) and representing individual European neurologists (principle of the former *European Neurological Society*). These articles also show how European neurology links with other societies in the field of neurosciences (psychiatry, neurosurgery, basic neurosciences), and with patient organizations, and how European neurology works with the World Health Organization and with the World Federation of Neurology. This first organizational part of this special issue is followed by a series of articles showing how concepts and the management of several neurological diseases have evolved over time. The recent SARS‐CoV‐2 pandemic has shown how important it is to remember lessons from another pandemic that occurred 1 century ago and killed more people than World War I: the Spanish flu.

We hope this special issue will reach its objective: helping European neurologists to have more knowledge of the past to better understand and prepare for the future.

## AUTHOR CONTRIBUTIONS

**Didier LEYS:** Conceptualization; writing – original draft. **Claudia Sommer:** Writing – review and editing. **Barbara Borroni:** Writing – review and editing. **Andrew Chan:** Writing – review and editing. **Mark Edwards:** Writing – review and editing. **Alessandro Tessitore:** Writing – review and editing. **Kristl Vonck:** Writing – review and editing.

## Data Availability

Data sharing not applicable to this article as no datasets were generated or analysed during the current study.

